# Generation and Characterization of Specific Monoclonal Antibodies and Nanobodies Directed Against the ATP-Gated Channel P2X4

**DOI:** 10.3389/fncel.2019.00498

**Published:** 2019-11-12

**Authors:** Philine Bergmann, Elvira Garcia de Paco, Björn Rissiek, Stephan Menzel, Gudrun Dubberke, Jennifer Hua, François Rassendren, Lauriane Ulmann, Friedrich Koch-Nolte

**Affiliations:** ^1^Institute of Immunology, University Medical Center Hamburg-Eppendorf, Hamburg, Germany; ^2^Institut de Génomique Fonctionnelle (IGF), University of Montpellier, CNRS, INSERM, Montpellier, France; ^3^Laboratoire d’Excellence Canaux Ioniques d’Intérêt Thérapeutique (LabEx ICST), Montpellier, France; ^4^Department of Neurology, University Medical Center Hamburg-Eppendorf, Hamburg, Germany

**Keywords:** purinergic receptors, monoclonal antibody, nanobody, immunoprecipitation, cytometry

## Abstract

The P2X4 channel is involved in different physiological and pathological conditions and functions in the nervous system. Despite the existence of several mouse models for which the expression of the gene was manipulated, there is still little information on the expression of the protein at the cellular level. In particular, supposedly specific available antibodies have often proved to recognize unrelated proteins in P2X4-deficient mice. Here, we used an *in vivo* DNA vaccine approach to generate a series of monoclonal antibodies and nanobodies specific for human, mouse, and rat P2X4 channels. We further characterized these antibodies and show that they solely recognize the native form of the proteins both in biochemical and cytometric applications. Some of these antibodies prove to specifically recognize P2X4 channels by immunostaining in brain or sensory ganglia slices, as well as at the cellular and subcellular levels. Due to their clonality, these different antibodies should represent versatile tools for further characterizing the cellular functions of P2X4 in the nervous system as well as at the periphery.

## Introduction

ATP is an extracellular signaling molecule that acts through two main families of plasma membrane receptors: the P2Y receptors, which are G protein-coupled receptors, and P2X receptors (P2XR), which are ATP-gated cationic channels (Burnstock, 2006). There are seven P2X subunits (P2X1-7) that associate to form homotrimeric or heterotrimeric receptors. In contrast to other ligand-gated channels, whose expression is mostly confined to the nervous system, P2XRs are expressed in numerous tissues and cell types (Surprenant and North, 2009). While some P2XRs are preferentially expressed in the nervous system (P2X2, P2X3), others are only found in peripheral tissue (P2X1) or are widely expressed (P2X4, P2X5, P2X7). Among all of the P2XRs, P2X4 shows the most widespread expression. It is found in diverse tissues, such as the brain, kidney, heart, lung, and salivary glands; it is expressed by many different cell types, including immune, epithelial, endothelial, and neuronal cells (Suurväli et al., 2017). Compared to other P2X receptors, P2X4 presents specific features. First, it is highly permeant to calcium; with a fractional calcium current close to 12–14%, P2X4 is as permeant to calcium as the NMDA receptors, and this is considered a major pathway for synaptic calcium influx (Egan and Khakh, 2004). Second, in resting cells, most P2X4 protein is localized in intracellular compartments along the endo-lysosomal pathway (Murrell-Lagnado and Frick, 2019). Signals that trigger the fusion of lysosomes with the plasma membrane bring a pool of intracellular P2X4 receptors to the cell surface. Third, P2X4 rapidly internalizes following its activation, possibly to protect the cell from calcium overload.

Despite recent advances, the pharmacology of P2X4 is still underdeveloped (Stokes et al., 2017). A few specific antagonists have been identified, showing species specificity and decent potencies. However, with a few exceptions, their efficacy *in vivo* is still poorly documented. Some insights into the physiological functions of P2X4 have been obtained through genetic ablation of its gene or down-regulation of its expression using RNA interference. These studies have implicated P2X4 in different pathologies (Tsuda et al., 2003; Sim et al., 2006; Yamamoto et al., 2006; Yang et al., 2014). A key feature of P2X4 is its expression in epithelial and endothelial cells, where luminal ATP triggers its activation. In endothelial blood vessel cells, shear stress due to high blood pressure evokes ATP release, which activates P2X4, which in turn triggers a calcium-dependent production of vasorelaxant nitric oxide (Yamamoto et al., 2006). Similarly, in lung epithelia, P2X4 activation is involved in mucus secretion (Winkelmann et al., 2019). Another key feature of P2X4 is its expression in different immune cells, such as myeloid cells and T lymphocytes. The functions of P2X4 are well documented in macrophages and microglia, where its activation triggers inflammatory and neuropathic pain, respectively (Ulmann et al., 2008, 2010). Of interest, while a strong P2X4 expression is observed in tissue-resident macrophages, it is not present in resting microglial cells. However, the expression of P2X4 is induced *de novo* in reactive microglia. In the CNS, the functions of P2X4 are still elusive. In BAC transgenic reporter mice, its expression was found to be sparse in the CNS, with a high level of expression in the hypothalamus, where it could control feeding behaviors (Xu et al., 2016). However, BAC transgenic reporter mice do not always accurately report on the actual expression of the gene of interest, as shown for the Genesat P2X7 eGFP-BAC reporter strain (Kaczmarek-Hajek et al., 2018).

Mapping P2X4 protein expression is still challenging. While there are two genetically modified mouse strains that allow *p2rx4* promotor activity to be followed: (1) a knock-in of the ß-galactosidase gene in the first exon; and (2) a BAC transgenic reporter mouse (Sim et al., 2006; Xu et al., 2016), these strains do not provide accurate information on the dynamics of receptor expression, as the half-lives of the receptor and the reporters likely differ. In addition, these reporter strains do not provide any information on the subcellular localization of the receptor. Cellular and biochemical characterization of proteins is facilitated by the use of antibodies. Regarding P2X4, several commercial and some homemade polyclonal antibodies are available. However, in our experience, the vast majority of these antibodies lack specificity, as they still show reactivity with proteins in P2X4-null mice. A considerable drawback of polyclonal antibodies is their inconsistency between different production lots, particularly when they originate from different immunized hosts (Roncador et al., 2016). This inconsistency presumably reflects the variability of the host immune response to a given immunogen. This is particularly problematic in the case of some commercial P2X4 antibodies for which validation of the specificity was performed on knockout mice samples for one given lot and then applied uniformly to other lots. An alternative to polyclonal antibodies are monoclonal antibodies, which because of their clonality, do not present variability issues and offer good reproducibility from lot to lot. However, because they recognize a single epitope, their signal intensity may be weaker and they may not work in all applications.

Here, we describe the generation and validation of a series of monoclonal rat antibodies and of recombinant llama nanobodies raised against either murine or human P2X4 in native conformation and demonstrate their utility for detecting P2X4 in living cells and fixed tissue and for immunoprecipitating P2X4 from undenatured cell extracts.

## Materials and Methods

### Bone Marrow-Derived Macrophage (BMDM) Culture

Bone marrow-derived macrophage (BMDMs) were obtained from bone marrow cells by flushing the femurs and tibias of 6- to 12-week-old mice. The bone marrow was homogenized, and the resulting fresh bone marrow cells were cultured in Glutamax-DMEM medium (Invitrogen) containing 100 U/ml penicillin and 100 μg/ml streptomycin (Invitrogen), supplemented with 10% fetal calf serum (FCS; Biowest) and 30% L929 cell-conditioned medium (LCCM). Four days after seeding the cells, fresh medium was added, and cells were incubated for an additional 4 days. To obtain the BMDM, the supernatants were discarded, and the attached cells were washed with 10 ml of sterile PBS. The macrophages were detached by cell scraping, counted, seeded, and cultivated in tissue culture plates for 12 h before any further experimental procedure.

### Mast Cell Degranulation Assay

Mast cells were obtained from the peritoneal cavity of C57BL/6 WT or P2X4^−/−^ mice by lavage with 5 ml cold PBS + EDTA (2 mM, Gibco). Cells were resuspended in RPMI medium containing 10% FCS, and aliquots were incubated for 10 min at 37°C in the presence or absence of 2 mM ATP (Sigma). Mast cells were identified by flow cytometry as CD11b^−^FcεR1^+^ using anti-CD11b (1/100, BioLegend, clone M1/70) and anti-FcεR1 (1/100, BioLegend, clone MAR-1) antibodies. ATP-induced degranulation of mast cells was evaluated by flow cytometry using antibodies against CD107a (1/100, BioLegend, clone 1D4B) and P2X4-specific monoclonal antibodies and nanobodies in order to detect the externalization of CD107a and P2X4 at the mast cell surface.

### Culture of Cell Lines and Transfections

Human Embryonic Kidney 293 cells (HEK) were cultured in Glutamax-DMEM medium (Invitrogen) containing 1% 100 U/ml penicillin and 100 μg/ml streptomycin (Invitrogen) and supplemented with 10% FCS (Biowest). Transfections of plasmids expressing the appropriate protein of interest (eGFP, P2X receptors, as specified in the results or figure legends) were performed using 3 μl per 35-mm dish of Lipofectamine 2000 (Life Technologies) according to the manufacturer’s recommendations.

### Immunofluorescence

BMDM cells were plated on coverslips and fixed in 1% paraformaldehyde for 5 min at RT. Cells were then washed in PBS and blocked in PBS, 3% BSA for 1 h and incubated for 1 h at RT with the primary antibody [rat anti-P2X4 (Nodu 246), 1/1,000]. After three washes, cells were incubated for 30 min at RT with a specific Alexa 488 goat anti-rat secondary antibody (1/2,000).

Mice were euthanized by peritoneal injection of pentobarbital (300 mg/kg). Blood was removed with intracardiac perfusion of PBS. L4, L5, and L6 DRG (dorsal root ganglion) and brain were placed in 4% paraformaldehyde in 0.1 M phosphate buffer pH 7.4 for 2 h. Tissues were cut into 30 μm sections with a vibratome, rinsed with PBS, and blocked with 10% goat serum diluted in a 0.1% Triton X-100 solution for 1 h. Monoclonal rat anti-P2X4 (Nodu 246) antibody was added overnight at 4°C. After rinsing, slices were incubated for 2 h with an Alexa488-conjugated goat anti-rat IgG secondary antibody (1/1,000). Primary and secondary antibodies were diluted in PBS with 0.1% Triton X-100. After rinsing, sections were mounted with Fluorescent Mounting medium (Dako) and observed on an AxioImager Z1 apotome (Zeiss).

### Western Blot

HEK cells were homogenized in lysis buffer (100 mM NaCl, 5 mM EDTA, 1% NP-40, 20 mM Hepes and complete protease inhibitor cocktail (Roche), pH 7.4). Lysates were clarified by centrifugation, and the protein concentration was determined using a protein assay kit (Bio-Rad). Proteins were separated on 12–4% NuPage precast gel (Life Technologies) and transferred onto a nitrocellulose membrane. The membrane was blocked with 5% non-fat dry milk/1% Tween20 in Tris-buffered saline (TBST) 1 h at RT. The membrane was then incubated o/n at 4°C with the appropriate antibody diluted in TBST: monoclonal rat anti-P2X4 (Nodu 246) or polyclonal rabbit anti-P2X4 antibody (1/500, Alomone Laboratory). After four washes in TBST, the membrane was probed with specific secondary antibodies conjugated to HRP (1/5,000) for 1 h at RT, revealed with SuperSignal West Pico Chemiluminescent Substrate (Pierce), and detected with a ChemiDoc™ Imaging System (Bio-Rad).

### Immunoprecipitation

Plasma membrane-enriched protein fractions were prepared from cultures of WT or P2X4 KO mouse BMDM. Cells were detached by cell scraping, counted, pelleted, and homogenized in a solubilization buffer [0.32 M sucrose, 10 mM Hepes, 2 mM EDTA, and complete protease inhibitor cocktail (Roche), pH 7.4] with 150 strokes of a Potter–Elvehjem homogenizer. Homogenates were centrifuged for 20 min at 1,264 rpm at 4°C to eliminate cell debris, and the supernatant was ultracentrifuged for 1 h at 70,000 rpm at 4°C. The resulting pellet was solubilized in Complexiolyte CL48 (Logopharm) supplemented with protease inhibitors (Roche) for 2 h at 4°C. Unsolubilized material (pellet; particles >336 S) was removed by centrifugation (30,000 rpm, 18 min, 4°C), transfected HEK cells were homogenized in lysis buffer [100 mM NaCl, 20 mM Hepes, 5 mM EDTA, 1% NP-40, and complete protease inhibitor cocktail (Roche), pH 7.4] 48 h after transfection. Lysates were clarified by centrifugation. The protein concentration of the lysates was determined using a protein assay kit (Bio-Rad). Freshly prepared solubilisates were incubated o/n at 4°C with Nodu 246 crosslinked to magnetic beads (Dynabeads, Invitrogen). The flow-through was discarded and the beads washed five times with wash buffer [CL48 diluted 1:4 in PBS and supplemented with complete protease inhibitor cocktail (Roche)], and sample buffer (Invitrogen) was added to separate the protein complexes from the beads. Proteins were resolved by SDS-PAGE, transferred to nitrocellulose membranes, and visualized using polyclonal rabbit anti-P2X4 (1/1,000, Alomone).

### Flow Cytometry

The expression of cell surface molecules was assessed by detaching HEK or BMDM cells by cell scraping and then counting and centrifuging them. They were then incubated for 20 min on ice with rat anti-P2X4 (Nodu 246) diluted in wash buffer (PBS supplemented with 1% BSA). After washing, the cells were probed with a fluorochrome-conjugated goat anti-rat IgG secondary antibody (1/200) for 20 min on ice. For intracellular staining of P2X4, the same protocol as for surface staining was employed, except that 3% saponin was added to the wash buffer in order to permeabilize the cells (permeabilization buffer). Bivalent Nb-rabbit IgG heavy-chain antibodies were used at 1/100 dilution, and bound antibodies were detected with Alexa 647-conjugated goat anti-rabbit IgG (1/200). The expression of P2X4 was analyzed by flow cytometry (FACSCalibur II or FACS Canto, Becton Dickinson). In some experiments (peritoneal cells, BMDM), cells were unstimulated or incubated at 37°C either with ATP (2 mM) or ATP (2 mM) and ivermectin (IVM, 3 μM, Sigma-Aldrich) for 10 min prior to FACS analysis.

### Targeting of the P2X4 Gene and Generation of Mutant Mice

Mice carrying a targeted null mutation of the P2X4 gene were described previously (Sim et al., 2006). Mice were housed under a standard 12 h light/dark cycle with food and water available *ad libitum*. Age varied between 6–12 weeks. APP/PS1; P2X4^−/−^ were obtained by breeding P2X4^−/−^ mice with the APPswe:PS1dE9 mice (Jackson Laboratory, stock# 034832). All procedures fully complied with French legislation (decree 87-848, October 19, 1987, and order, April 19, 1988), which implements the European directive (86/609/EEC) on research involving laboratory animals, and were performed according to the requirements of CNRS ethical standards.

### Generation of P2X4-Specific Monoclonal Antibodies and Nanobodies

Rats and llamas were immunized with P2X4^Y378F^-encoding mammalian expression vectors conjugated to gold particles by ballistic intradermal immunization, essentially as described previously (Möller et al., 2007; Eden et al., 2018). Spleen and lymph nodes were prepared from rats 4 days after the final boost; peripheral blood leukocytes were prepared from llamas 7 and 10 days after the final boost. Spleen and lymph node cells were fused to Sp2/0 myeloma cells, and hybridoma cells were selected in HAT medium in 96-well-plates. The VHH repertoire was PCR-amplified from cDNA of peripheral blood leukocytes and cloned into the pHEN2 phage display vector. The phage library was subjected to two rounds of panning on untransfected and P2X4^Y378F^-transfected HEK cells. Sequencing of the VHH coding region identified families of enriched clones. The VHH coding region was cloned into the mammalian pCSE2.5 expression vector upstream of the hinge, CH2, and CH3 domains of rabbit IgG. Supernatants containing chimeric Nb-rabbit IgG heavy chain antibodies were collected 6 days after transfection, and supernatants from hybridoma cells 12–16 days after cell fusion. Cell supernatants were analyzed for the presence of P2X4-specific antibodies by immunofluorescence microscopy using CHO cells 24 h after co-transfection with expression vectors for P2X4 and GFP-NLS (nuclear localization signal) fusion protein. Bound antibodies were detected with PE-conjugated rat or rabbit IgG-specific secondary antibodies. P2X4-specific antibodies were purified by affinity chromatography on protein A-sepharose and conjugated *via* NH2-esters to Alexa fluorescent dyes. All procedures fully complied with German legislation, which implements the European directive (86/609/EEC) on research involving laboratory animals, and were performed according to the requirements of the University Medical Center Hamburg Eppendorf.

## Results

### Generation of Rat Monoclonal Antibodies Against P2X4 Receptors

**Figure 1:**
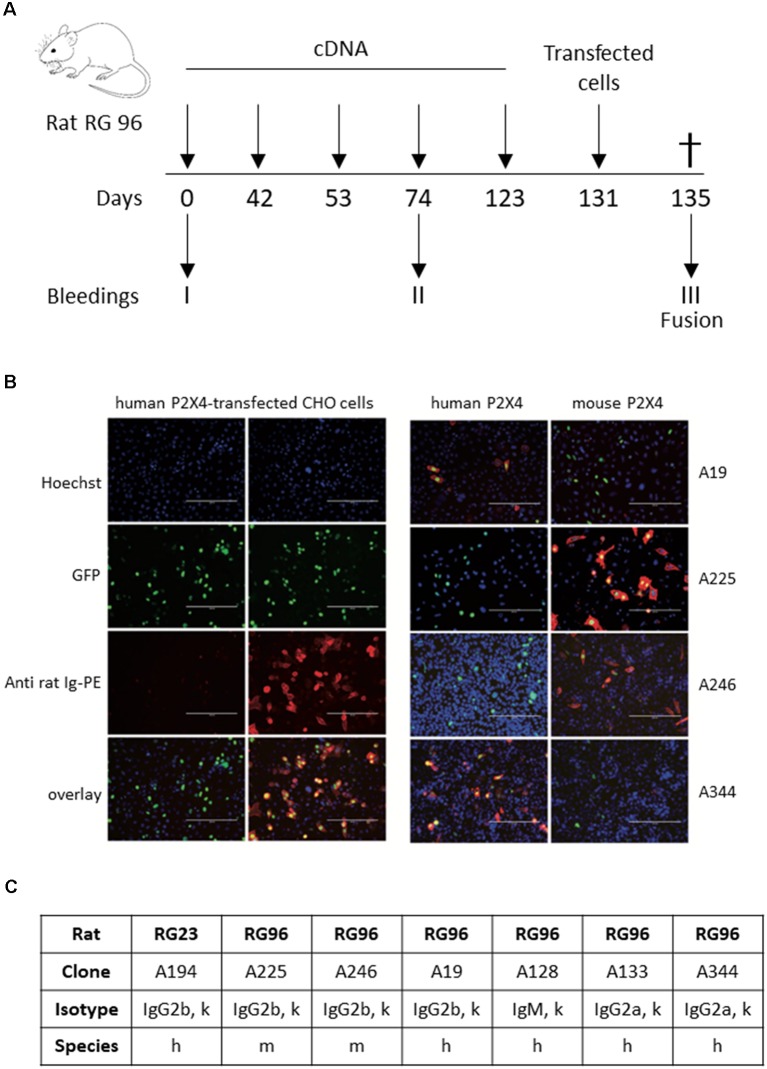
Identification of P2X4-specific monoclonal antibodies. **(A)** Schematic diagram of the rat immunization protocol. Rats were immunized five times by trans-dermic biolistic gene delivery (six shots per immunization) with expression vectors encoding either mouse WT P2X4 (rat RG23) or mouse and human P2X4-Y378F (rat RG96). Each rat received a final injection of CHO cells expressing either mouse P2X4 or a mixture of mouse and human P2X4-Y378F. Pre-immune sera were drawn at day 0 and immune sera at day 74, prior to the final boost at day 123. Terminal bleeding was performed on day 131, followed by cell fusion and hybridoma selection. **(B)** Pre-immune sera, immune sera (left panels, dilution 1/1,600) of rat RG96 and hybridoma supernatants derived from RG96 (right panels) were tested for reactivity with CHO-cells transiently co-transfected with expression constructs for nuclear GFP and either human P2X4 or mouse P2X4-Y378F, as indicated. Different hybridoma show specificity toward human or mouse P2X4 receptors. Scale bar: 200 μm. **(C)** Specific characteristics of the different identified hybridoma.

A combination of genetic and full-length protein immunization was used to maximize the chance of obtaining antibodies recognizing the native form of the receptor (Adriouch et al., 2005; Koch-Nolte et al., 2019). Gene gun immunization was used to immunize two rats according to the protocol shown in Figure 1A, either with a cDNA encoding mouse P2X4-Y378F (Rat RG23) or a mixture of mouse and human P2X4-Y378F cDNAs (Rat RG96), respectively. A final boost was performed using HEK cells transiently expressing either mouse P2X4 or both human and mouse P2X4. After terminal bleeding and fusion of splenocytes to Sp2/0 myeloma cells, individual hybridoma cells were selected by limit dilution, and the presence of antibodies recognizing P2X4 in the supernatant was tested by immunocytochemistry on CHO cells transiently transfected with either human or mouse P2X4-Y378F. Figure 1B shows examples of immune staining obtained using the supernatant of different hybridomas originating from rat RG96 (right panel) and pre-immune and immune sera from the same animal (left panel). The results show that two clones (Nodu 225 and Nodu 246) bind only mouse P2X4, while the other two (Nodu 19 and Nodu 344) only recognize human P2X4. The characteristics of the different hybridomas obtained are indicated in Figure 1C.

**Figure 2:**
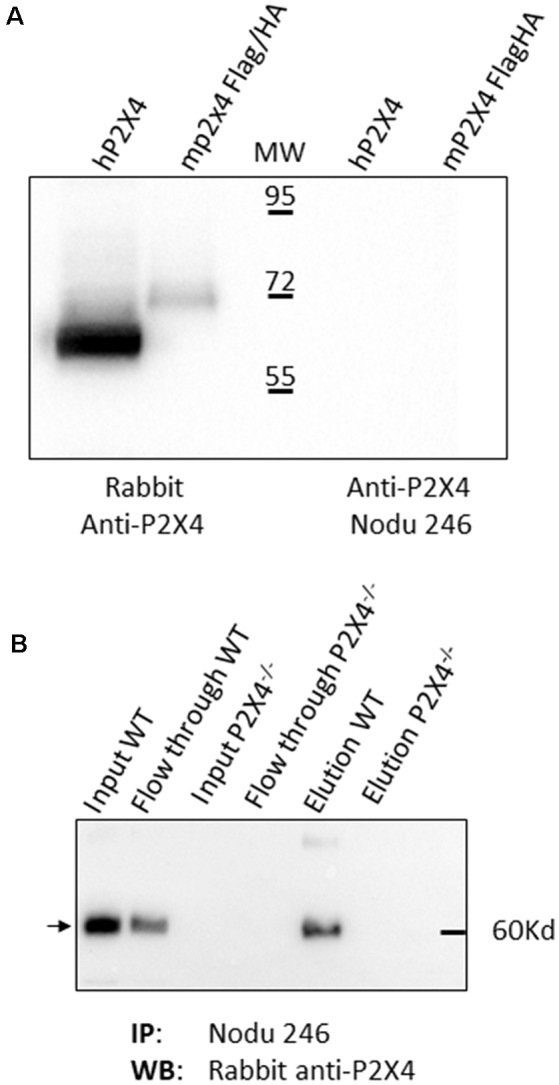
Biochemical properties of Nodu 246 hybridoma. **(A)** Recognition of denatured human and mouse P2X4 receptors by mAb Nodu 246 was assessed by Western blotting. Protein extracts from a HEK cell line stably expressing the human P2X4 and HEK cells transiently transfected with an expression construct for Flag/HA-tagged mouse P2X4 were analyzed by Western blot after electrophoresis of cell lysates on an SDS-polyacrylamide gel. Blots were incubated either with a rabbit polyclonal anti-P2X4 peptide antibody (Alomone) or with rat monoclonal antibody Nodu 246. Bound antibodies were revealed with peroxidase-conjugated anti-rabbit IgG or anti-rat IgG. The monoclonal mAb Nodu 246 evidently does not recognize the denatured forms of human and mouse P2X4 (right panel), as opposed to the rabbit polyclonal anti-peptide antibody (left panel). **(B)** P2X4 in mouse WT and P2X4 KO bone marrow-derived macrophage (BMDM) was specifically immunoprecipitated with Nodu 246. Immunoprecipitated proteins were separated by electrophoresis on SDS-polyacrylamide gels and immunoblotted with a polyclonal rabbit anti-P2X4 peptide antibody (Alomone). The same protein extracts were loaded on both panels. Note the higher apparent molecular weight of the Flag/HA-tagged mouse P2X4 compared to human P2X4.

We next fully characterized the Nodu 246 monoclonal antibody, which specifically recognizes mouse P2X4, by immunostaining. We first evaluated its capacities to recognize native or denatured forms of the receptor *via* biochemical assay. We analyzed whether Nodu 246 could detect mouse P2X4 in a typical Western blotting experiment. Protein extracts from HEK cells transiently expressing either human P2X4 or Flag/HA-tagged mouse P2X4 were separated by SDS-PAGE and transferred to a nitrocellulose membrane. The blot was incubated sequentially with Nodu 246 and peroxidase-conjugated anti-rat IgG. Bound antibodies were visualized by enhanced chemiluminescence (Figure 2A). The results show that Nodu 246 is unable to detect denatured human or mouse P2X4, while a commercial polyclonal rabbit-antibody raised against a peptide conserved in mouse and human P2X4 reacts with protein bands at the expected molecular weight of ~60 kD. These results indicate that Nodu 246 only recognizes mouse P2X4 in its native conformation. We next tested whether Nodu 246 could immunoprecipitate endogenous P2X4 from mouse BMDMs. Immunoprecipitation of P2X4 was performed on BMDM from either WT or P2X4-null mice. Immunoprecipitates were analyzed for the presence of mouse P2X4 by Western blotting using commercial P2X4 peptide-specific rabbit antibodies (Figure 2B). The results show that Nodu 246 efficiently immunoprecipitated P2X4 from WT BMDM but not from P2X4^−/−^ cells. Similar experiments performed on HEK cells transiently expressing human P2X4 indicate that Nodu 246 is unable to immunoprecipitate the human receptor (not shown), confirming the results obtained by immunocytochemistry. Nodu 246 did not cross-react with human and mouse P2X7 receptors (data not shown); cross-reactivity to other subunits was not tested.

**Figure 3:**
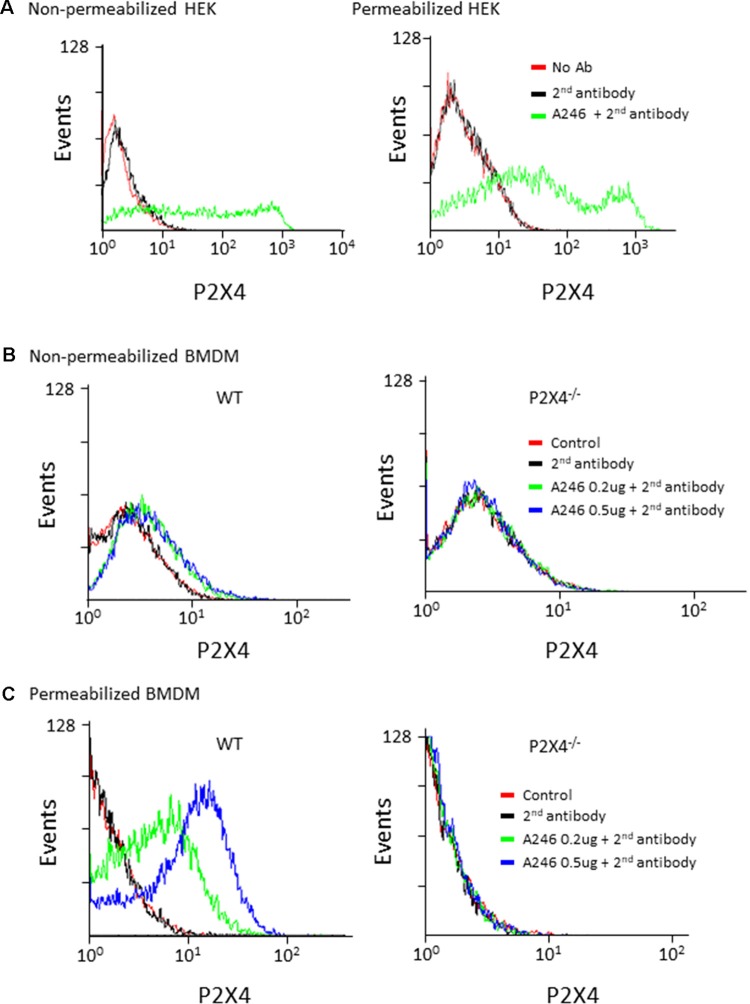
Flow cytometry analysis of mouse P2X4 expression using Nodu 246. **(A)** Cell surface and intracellular expression of mouse P2X4 by P2X4-transfected and non-transfected HEK cells was assessed by flow cytometry after sequential incubation of cells with rat mAb Nodu 246 followed by a secondary PE-conjugated anti-rat-IgG antibody (2nd antibody), in non-permeabilized or permeabilized cells. Similar experiments were performed with BMDM obtained from WT and P2X4^−/−^ mice. Cells were either non-permeabilized **(B)** or permeabilized **(C)**. Note that permeabilization greatly enhanced the detection of mouse P2X4 in BMDM, further supporting the intracellular localization of the native receptor.

Nodu 246 was produced by cDNA immunization, a strategy aimed at producing antibodies that recognize native extracellular epitopes of membrane proteins (Möller et al., 2007; Eden et al., 2018). We thus analyzed the performance of Nodu 246 in flow cytometry experiments. We first used HEK cells transiently expressing mouse P2X4. As shown in Figure 3A, when tested on non-permeabilized HEK cells expressing P2X4, a specific fluorescence signal was present, although the number of events was relatively small and covered a large range of intensities. However, after permeabilization, the overall fluorescence signal increased, suggesting that even in transfected HEK cells, most P2X4 receptors are localized intracellularly. Similarly, in non-permeabilized mouse BMDM, Nodu 246 generated a weak fluorescent signal that was strongly enhanced upon permeabilization. No specific fluorescence signal was observed when the same experiments were performed on P2X4^−/−^ BMDM (Figures 3B,C).

**Figure 4:**
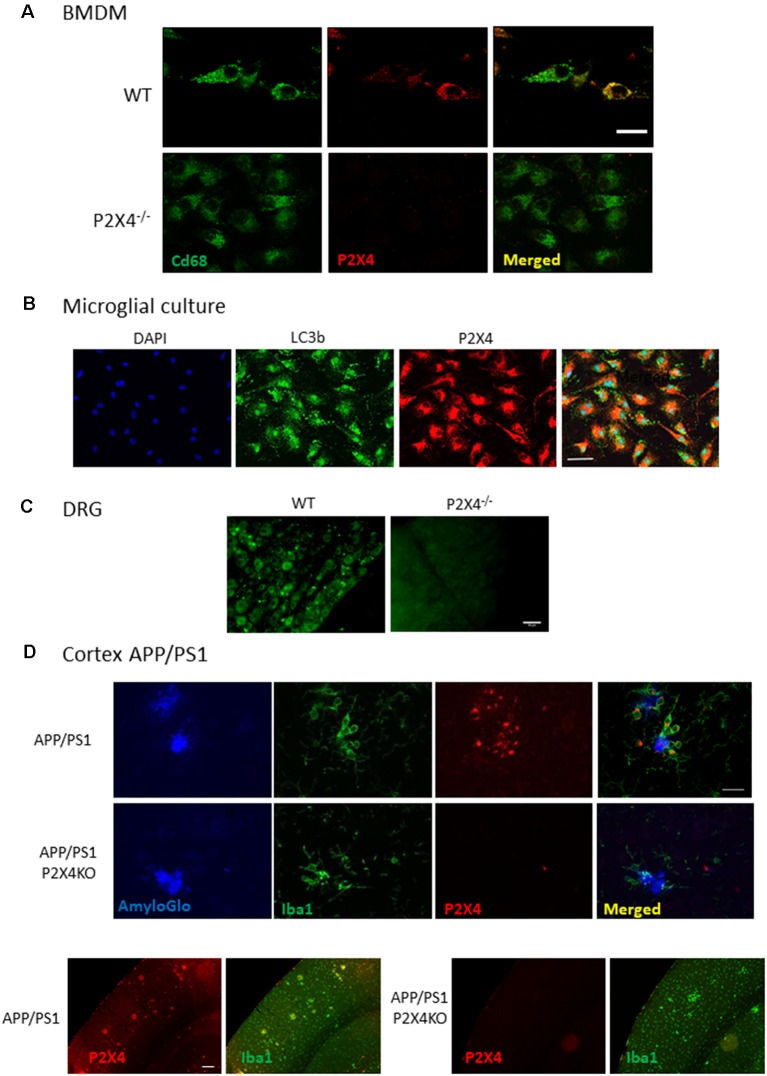
Immunocyto- and -histochemistry with Nodu 246. **(A)** Co-immunostaining of WT and P2X4^−/−^ BMDM with Nodu 246 and the lysosomal marker CD68. Scale bar: 5 μm. **(B)** Co-immunostaining of WT and P2X4^−/−^ cultured mouse microglia using the rat-monoclonal antibody Nodu 246 and the lysosomal marker LC3b. Scale bar: 10 μm. **(C)** Immunostaining of dorsal root ganglion (DRG) of WT and P2X4^−/−^ DRG. Scale bar: 50 μm. **(D)** Top panel, co-immunostaining of microglial cells (green, Iba1) and P2X4 (red, Nodu 246) and amyloid plaques (blue, AmyloGlo) in the cortex of brains from APP/PS1 and APP/PS1; P2X4^−/−^ mice. P2X4 is localized in the intracellular compartments of activated microglia clustered around amyloid deposits. Scale bar 20 μm. Bottom panel, low magnification of the cortical region of APP/PS1 and APP/PS1; P2X4^−/−^ mice stained with anti-Iba1 (green) and Nodu 246 (red). Note the dim P2X4 immunostaining in cortical neurons. Scale bar 50 μm.

Next, we assessed the immunostaining performances of Nodu 246 on fixed primary cultured cells and tissue sections (Figures 4A,B). In a primary culture of permeabilized BMDM or microglia, the Nodu 246 signal was localized in intracellular compartments and co-localized with either CD68 or LC3b, suggesting an intracellular localization of P2X4 in the endo-lysosomal compartment (Figures 4A,B). No staining was observed in BMDM from P2X4^−/−^ mice, supporting the specificity of the Nodu 246 antibody. Specificity was also demonstrated in tissue sections of DRG (Figure 4C), showing, as previously reported, that P2X4 is expressed in a subset of sensory neurons (Lalisse et al., 2018). Specificity was also tested in the cortex of 12-month-old APP/PS1 mice (Figure 4D); non-specific staining was not observed in brain sections from P2X4^−/−^ mice, while in the APP/PS1 mice, P2X4 was localized in specific intracellular compartments of reactive microglia clustered around amyloid deposits but not in microglia distant from the plaques. Dim immunostaining was also present in cortical neurons.

The specificity of Nodu 246 was not tested on recombinantly expressed P2X subunits. However, in P2X4-deficient mice, Nodu 246 did not stain macrophages, DRG, and brain sections, in which expression of all P2X subunits is well characterized, supporting the specificity of the Nodu 246.

### Generation of Nanobodies Against P2X4 Receptors

**Figure 5:**
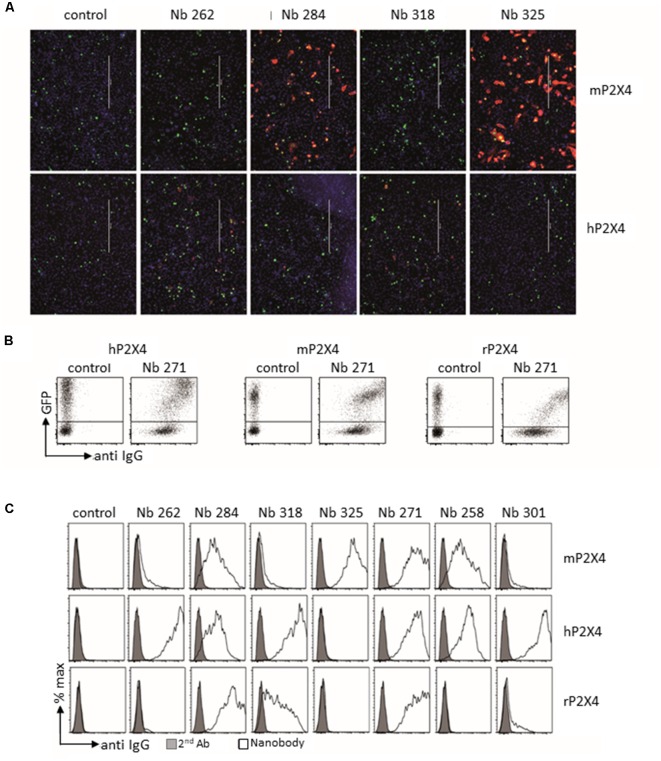
Analysis of cloned nanobodies against mouse or human P2X4. **(A)** Immunofluorescence analysis of the reactivities of monovalent his6x-c-myc-tagged nanobodies with non-permeabilized CHO cells transiently co-transfected with GFP and either mouse (top panel) or human (bottom panel) P2X4 Y378F. Bound nanobodies were detected by sequential staining with a C-myc tag-specific mouse mAb followed by PE-conjugated rabbit anti-mouse IgG. Scale bar: 400 μm. **(B,C)** Flow cytometry analysis of the binding specificity of the different selected nanobodies. HEK cells were co-transfected with GFP and either human, mouse, or rat P2X4 Y378F. The FACS analyses in **(B,C)** were performed on transiently transfected HEK cells using bivalent Nb-rabbit IgG heavy chain antibodies. Bound antibodies were detected with Alexa 647-conjugated goat anti-rabbit IgG. **(B)** Example of results obtained with Nb 271, which binds all tested P2X4 receptors. **(C)** Binding specificity of the different selected nanobodies. Gray graphs correspond to control staining using only the secondary antibody. Nb isotype corresponds to a control with an unrelated isotype nanobody. The different staining protocols used to account for the stronger labeling intensity of human P2X4 transfected cells in **(B,C)** vs. **(A)**. The relative staining intensities of the nanobodies are the same in panels **(A,B)**, i.e., for mP2X4: 325 > 284 > 318 = 262 and for hP2X4: 262 > 318 > 284 > 325.

Llama immunization was performed using a similar strategy as for rat, above. Two llamas were immunized four times at 2-week intervals using cDNA expression vectors encoding either mouse or human P2X4-Y378F, followed by a final boost with P2X4-transfected HEK cells. Blood was sampled 4 and 8 days following the last boost, and the VHH repertoire of vascular B cells was PCR-amplified and used for phage library construction. Positive clones were selected through two cycles of phage panning on HEK cells stably expressing recombinant human or mouse P2X4. Selected clones were produced as secreted periplasmic proteins carrying a C-terminal His6x-c-myc tag in *E. coli*. The specificity of the recombinant nanobodies was tested by immunofluorescent staining of CHO cells transiently co-transfected with expression vectors encoding human or mouse P2X4 and nuclear GFP. Bound nanobodies were detected using fluorochrome-conjugated monoclonal antibody directed against c-myc. Figure 5A shows representative results obtained for four different clones out of seven compared to an irrelevant nanobody. The seven VHH were subcloned into a eukaryotic expression vector so as to replace the C-terminal His6x-c-myc tag by the hinge, CH2, and CH3 domains of rabbit IgG, providing the basis for generating a bivalent nanobody-rabbit IgG heavy chain antibody (Rb-hcAb), which allows for increased avidity and easy production in eukaryotic cells. The species specificity of the different nanobodies produced as Rb-hcAb was assessed using flow cytometry of HEK cells transiently co-transfected with cDNA expression vectors encoding eGFP and either human, mouse, or rat P2X4 Y378F. Figure 5B shows an example of FACS sorting of HEK cells expressing either human, mouse, or rat P2X4, using GFP and a fluorochrome-conjugated secondary anti-rabbit-IgG antibody for detecting the Rb-hcAb constructed with nanobody 271 (Nb 271). Figure 5C shows the species specificity of the seven Nbs after gating on GFP-positive cells compared to cells incubated with the secondary antibody only. Two clones recognize human, mouse, and rat P2X4 (Nb 271 and Nb 284), two react only with human P2X4 (Nb 262, Nb 301), one reacts with both human and mouse P2X4 (Nb 258), one reacts with both human and rat P2X4 (Nb 318), and one reacts only with mouse P2X4 (Nb 325). Specificity was also tested on cells transfected with expression vectors for human and mouse P2X7 and P2X1. With the exception of Nb 301, which also recognizes both human and mouse P2X7, the other Nbs did not show any cross-reactivity with P2X7 or P2X1 (data not shown). Specificity against other P2X subunits was not tested.

**Figure 6:**
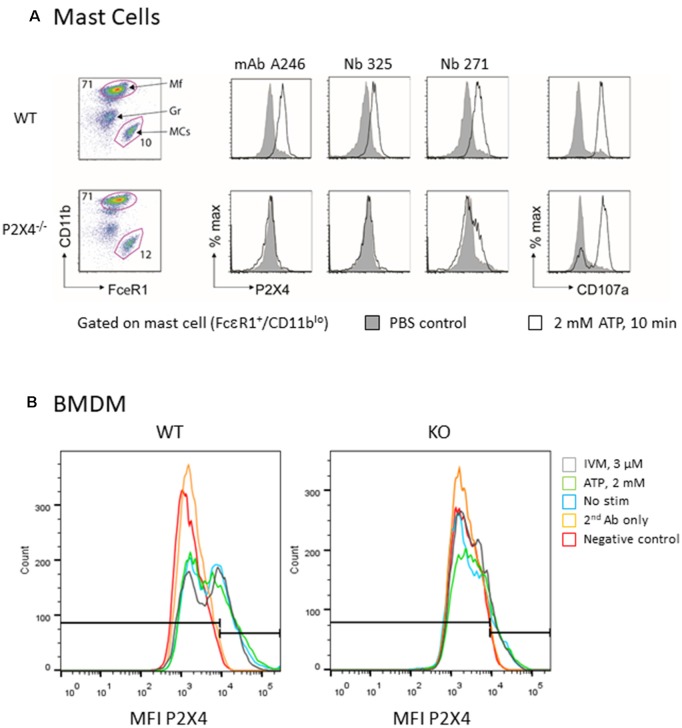
Flow cytometry analysis of native mouse P2X4 expressed by peritoneal mast cells and BMDM. **(A)** Peritoneal cells from WT and P2X4^−/−^ were stimulated (white) or not (gray) for 10 min with 2 mM ATP at 37°C. Cells were washed and incubated at 4°C with anti-CD11b-PerCO and anti-FcεR1-PE as well as with various antibodies directed against P2X4 and appropriate secondary antibodies (AF647 or AF488). CD107a (LAMP1-FITC) was used as a control experiment. Mast cells were identified as CD11b low, FcεR1+. mAb Nodu 246: rat monoclonal antibody; Nb 325, Nb 271: Nanobodies. **(B)** Similar flow cytometry experiments of WT and P2X4^−/−^ BMDM using Nb 271 RbhcAb. Cells were stimulated as above with 2 mM ATP (ATP, green), 3 μM ivermectin (IVM, gray), or not stimulated (nanobody, blue). Negative controls were secondary antibody only (secondary antibody, orange) and unlabeled cells (Neg, red).

Treatment of mast cells with ATP reportedly evokes degranulation and lysosomal fusion with the plasma membrane (Wareham and Seward, 2016). We assessed whether the treatment of mouse mast cells with ATP induces exposure of P2X4 on the plasma membrane. Lysosomal fusion with the plasma membrane was visualized by flow cytometry using a fluorochrome-conjugated monoclonal antibody that recognizes the lysosomal protein Lamp1/CD107a (Figure 6). The results confirm that ATP stimulation strongly enhances the detection of Lamp1 in both WT and P2X4^−/−^ mast cells, indicating a lysosomal fusion with the plasma membrane and exposure of Lamp1 at the cell surface. Detection by Nb 271 and 325 of endogenous P2X4 expressed by the peritoneal mast and BMDM cells was also analyzed by cytometry (Figure 6). A similar experiment was performed with BMDM from WT and P2X4^−/−^ using Nb271-rbhcAb. In this case, cells were either unstimulated or incubated, either with ATP (2 mM) or ATP and IVM (3 μM), for 10 min as above. As shown in Figure 6B, a specific signal was observed in WT but not in P2X4^−/−^ BMDM. In contrast to peritoneal mast cells, a low level of P2X4 could clearly be detected on BMDM, even in the absence of ATP or IVM stimulation.

## Discussion

Antibodies are unique biological tools for characterizing protein expression, cellular distribution, and functions. There is a great diversity of antibody types, modes of immunization, species, and clonality, and assessing and validating their specificity is a major issue in the field (Baker, 2015; Roncador et al., 2016). This is particularly true for polyclonal antibodies, which are produced in limited amounts and require new validation for each new batch, assuming that batches correspond to different donors. Clonal antibodies such as monoclonal antibodies or nanobodies have the advantage of being unlimited, since they are either produced by immortalized hybridoma or cloned in eukaryotic expression vectors, respectively. This allows full characterization of their specificity and, once validated, the assurance of their reproducibility.

For different reasons, raising specific antibodies against transmembrane receptors is difficult. First, since these proteins contain one or more hydrophobic transmembrane domains embedded in the membrane leaflet, the purification of native membrane proteins for immunization is difficult. Second, producing recombinant membrane proteins in their native conformation is challenging. Thirdly, immunization with synthetic peptides derived from the amino acid sequence of a transmembrane protein does not ensure proper folding corresponding to that of the native protein. Resulting antibodies often do not recognize their target protein in native conformation. It is therefore important to improve the experimental design for generating membrane protein antibodies, leading to the generation of antibodies with increased specificity towards native protein. In this study, we used a specific strategy to generate monoclonal antibodies and nanobodies recognizing native P2X4, a transmembrane ATP-gated channel. Animals were immunized through cDNA immunization, an approach that uses a repetitive injection of a eukaryotic expression plasmid encoding P2X4. This strategy allows *in vivo* transfection of cells of the immunized host, which will express the foreign protein in its native conformation and trigger the specific immune response (Möller et al., 2007; Eden et al., 2018). Because P2X4 receptors are normally intracellularly localized in lysosomes, we immunized with an expression vector encoding the Y378F P2X4 mutant, that is unable to internalize (Qureshi et al., 2007), instead of the WT receptor. Downstream characterization demonstrates that this strategy was highly efficient in generating antibodies recognizing the extracellular epitope of the native protein in both rats and llamas. All antibodies identified in this study only recognize P2X4 from mouse, rat, and/or human in their native conformation but not in their denatured form. This is likely due to the screening and selection strategies used for the identification of positives clones, which in both cases rely on binding of the antibody to the cell expressing the receptors. It is possible that a screening strategy based on denatured protein would have led to the identification of different clones with other specificities.

Antibodies targeting the extracellular region of receptors represent potential pharmacological tools for modulating their functions (Koch-Nolte et al., 2019). This is particularly true for nanobodies, which, owing to their small size and long CDR3 loops, can often access ligand-binding pockets and thus either activate or block receptors (De Genst et al., 2006; Stortelers et al., 2018). Concerning P2X receptors, blocking antibodies and nanobodies have been generated for mouse and human P2X7 (Danquah et al., 2016). These nanobodies show therapeutic potential, as they can reduce P2X7-evoked inflammation *in vivo*. A recent study reported the generation of a panel of antibodies that modulate human and mouse P2X4 receptors positively and negatively (Williams et al., 2019). These antibodies were raised against purified recombinant P2X4 proteins using either page display screening or immunization in rats and were further engineered to improve their biological activities. While these antibodies can reverse neuropathic pain when systemically administered in animal models, their biochemical and immunostaining properties were not reported.

Although the biological properties of all the antibodies identified in this study have not been deeply investigated, neither Nodu 246 nor Nb 284 show any blocking or activating properties (data not shown). Different factors can explain this lack of biological activity. First, the Y378F P2X4 mutant was used for immunization. Although this mutant shows no obvious altered sensitivity to ATP compared to wild type receptors, its constant presence at the plasma membrane may result in its inactivation by resting extracellular ATP concentrations. This inactivated conformation may bury the ATP-binding pocket region and its immunogenic potential. Alternatively, P2X4 receptors are heavily glycosylated compared to P2X7; this can act as a shield, preventing the antigenic regions of the binding pocket from being exposed during immunization. Further characterization of the binding epitopes of the antibodies would be required to achieve a better understanding of the lack of pharmacological effect of these antibodies and, potentially, to design new immunization strategies.

In addition to the production of highly specific monoclonal antibodies against P2X4 receptor, our results provide a methodological framework for generating antibodies against other membrane proteins that has widespread potential applications.

## Data Availability Statement

Genebank accession numbers for the different cDNAs used in this study are: Y07684.1 (human P2X4), AF089751.1 (mouse P2X4) and X87763.1 (rat P2X4).

## Ethics Statement

The animal study was reviewed and approved by the animal welfare commission (Amt für Verbraucherschutz, Lebensmittelsicherheit und Veterinärwesen Hamburg, A8a/694).

## Author Contributions

FK-N and FR designed the study. PB, BR, SM, EG, GD, JH, and LU performed the experiments. LU, FK-N, and FR wrote the manuscript.

## Conflict of Interest

The authors declare that the research was conducted in the absence of any commercial or financial relationships that could be construed as a potential conflict of interest.
